# Roles of Gut Microbiota and Metabolites in Pathogenesis of Functional Constipation

**DOI:** 10.1155/2021/5560310

**Published:** 2021-09-21

**Authors:** Jun-Ke Wang, Shu-Kun Yao

**Affiliations:** ^1^Department of Gastroenterology, Peking Union Medical College and Chinese Academy of Medical Sciences, China-Japan Friendship Hospital, Beijing 100029, China; ^2^Department of Gastroenterology, China-Japan Friendship Hospital, Beijing 100029, China

## Abstract

Functional constipation (FC), a condition characterized by heterogeneous symptoms (infrequent bowel movements, hard stools, excessive straining, or a sense of incomplete evacuation), is prevalent over the world. It is a multifactorial disorder and can be categorized into four subgroups according to different pathological mechanisms: normal transit constipation (NTC), slow transit constipation (STC), defecatory disorders (DD), and mixed type. Recently, growing evidence from human and animals has pointed that there was a strong association between gut microbiota and FC based on the brain-gut-microbiome axis. Studies have reported that the main characteristics of gut microbiota in FC patients were the relative decrease of beneficial bacteria such as *Lactobacillus* and *Bifidobacterium*, the relative increase of potential pathogens, and the reduced species richness. Gut microbiota can modulate gut functions through the metabolites of bacterial fermentation, among which short-chain fatty acids (SCFAs), secondary bile salts (BAs), and methane occupied more important positions and could trigger the release of gut hormones from enteroendocrine cells (EECs), such as 5-hydroxytryptamine (5-HT), peptide YY (PYY), and glucagon-like peptide-1 (GLP-1). Subsequently, these gut hormones can influence gut sensation, secretion, and motility, primarily through activating specific receptors distributed on smooth muscle cells, enteric neurons, and epithelial cells. However, research findings were inconsistent and even conflicting, which may be partially due to various confounding factors. Future studies should take the associated confounders into consideration and adopt multiomics research strategies to obtain more complete conclusions and to provide reliable theoretical support for exploring new therapeutic targets.

## 1. Introduction

Constipation is a common syndrome characterized by bowel symptoms (infrequent bowel movements, hard stools, excessive straining, or a sense of incomplete evacuation) with reported prevalence ranging from 2.6% to 26.9% (an average of 16%) in the general adult population and from 0.7% to 29.0% in children worldwide [1–4]. It may occur either primarily or secondarily to other underlying conditions (e.g., mechanical obstruction, metabolic conditions, neuropathies, and depression) [1, 2, 5]. Primary constipation mainly includes functional constipation (FC) and constipation-predominant irritable bowel syndrome (IBS-C). FC is one of the most frequent functional gastrointestinal disorders (FGIDs) in the world, which is diagnosed according to Rome IV criteria, standardized and more expansive consensus criteria, in most current studies [6, 7], and can be classified into four subgroups based on the pathophysiology: normal transit constipation (NTC), slow transit constipation (STC), defecatory disorders (DD), and mixed type [1, 7, 8].

FC is a multifactorial disorder. Genetic predisposition, increasing age, female sex, lower social economic status and parental education rates, less self-reported physical activity, stressful life events, physical and sexual abuse, and psychological factors are common risk factors [2, 6, 9, 10]. Moreover, different pathophysiologic mechanisms have been reported between different subgroups. Patients with NTC only have subjective symptoms of constipation and the precise pathophysiology is unknown [11]. Colonic sensorimotor dysfunction, primarily due to reduction in colonic intrinsic nerves and interstitial cells of Cajal, changes of signals between the central nervous system (CNS) and enteric nervous system (ENS), and impaired smooth muscles, usually underlies STC [7, 8, 10–13]. DD mainly develop because of pelvic floor dysfunction during defecation, such as reduced rectal propulsive forces and/or increased resistance to evacuation [10, 11]. However, although existing findings have been obtained, the pathophysiology of FC is still not fully elucidated and these subtypes may overlap each other, making the mechanisms more complicated.

Gut microbiota, a complex community of microbes colonized in human body, involves in important physiological functions of the host, and gut dysbiosis may contribute to the occurrence and development of diseases due to the existence of brain-gut-microbiome axis [14–17]. Recently, several studies have investigated alterations in the composition of gut microbiota and possible microbial mechanisms associated with FC, especially several gut metabolites and hormones including short-chain fatty acids (SCFAs), secondary bile salts (BAs), methane, and 5-hydroxytryptamine (5-HT) [18–21]. These metabolites can affect gut motility and secretion of FC patients through activating corresponding receptors distributed in some enteroendocrine cells (EECs), enterochromaffin cells (ECCs), and neuronal cells to synthesize and release bioactive compounds, such as peptides and neurotransmitters. However, consensus has not been reached in most conditions. In this review, we aim to summarize the current evidence regarding the alterations of gut microbiota and possible roles of the microbial metabolites in the pathophysiology of FC. The discussion will throw considerable light on a new dimension in understanding the pathophysiology and management of FC.

## 2. Brain-Gut-Microbiome Axis and FC

**Figure 1:**
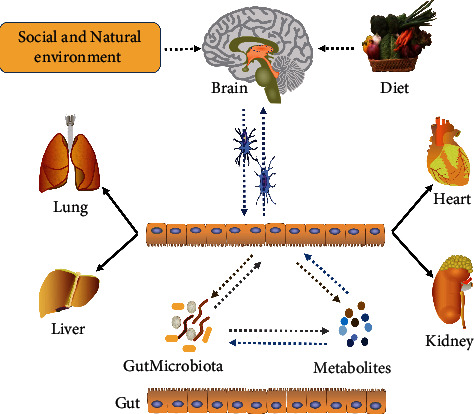
Interactions between diet and brain-gut-microbiome (including gut microbiota as well as metabolites). On the one hand, exposed to social pressure and natural environment, the host brain can regulate the gut functions (i.e., sensation, motility, and secretion) through nerve conductions and then influence the growth of gut microbiota, and vice versa. On the other hand, the food residues generated via digestion are subsequently fermented by gut microbiota, which can produce some metabolites; in turn, these bioactive metabolites can affect both microbiota and host gut and further send signals to the brain. Taken together, under healthy conditions, this intricate network remains in balance. In contrast, once the balance is broken, discomfort and even diseases will follow, including neurologic, respiratory, metabolic, hepatic, and cardiovascular illnesses.

Several trillion microbial cells live in the human gut, which influence the host immune response, protection against pathogen overgrowth, host-cell proliferation and vascularization, intestinal endocrine functions, and neurologic signaling as well as energy biogenesis [22]. Most functions are mediated by multiple biologically important molecules produced during the metabolism of food and xenobiotics, such as vitamins, neurotransmitters, SCFAs, secondary BAs, choline metabolites, phenols, phenol derivatives, terpenoids, polyamines, lipids, and hormones, which may contribute to the host metabolic phenotype and hence to disease risk [17, 23–25]. To be specific, driven by the natural and social environment, diet, brain (CNS), gut, and microbiota as well as substances derived from gut or microbiota formed a complex and bidirectional network interacting with each other (Figure 1), which has been proposed as the brain-gut-microbiome axis recently [17]. If there is a perturbation at any level of this axis, the homeostasis of the organism will be disturbed, which may lead to the occurrence and development of diseases, such as neurologic, respiratory, metabolic, hepatic, and cardiovascular illnesses [17, 22, 25–28]. Given that gut microbiota and metabolites act as “transfer station” in this axis, it is necessary to explore the precise microbial mechanisms in the pathogenesis of disorders.

From the perspective of the brain-gut-microbiome axis, a series of studies have reported that children and adults with FC experienced more negative events and usually had an abnormal mental state compared with healthy individuals, suggesting that psychological factors, specific psychological traits (anxiety and depression), and stressful life events might contribute to FC via triggering gut dysfunction [29–32]. Intriguingly, studies using functional MRI also reported that patients with FC showed different patterns of brain processing in response to rectal distention compared with healthy controls [12, 33]. Meanwhile, alterations of gut microbiota may be involved. Currently, several lines of evidence from human and animals have pointed that there was a strong association between gut microbiota and FC. Firstly, microbial treatments, such as prebiotics, probiotics, synbiotics, and fecal microbiota transplantation (FMT), can improve the clinical symptoms of some patients who suffered from FC with few adverse effects, including defecation frequency, stool consistence, and constipation-related discomfort (e.g., bloating and abdominal pain) [34–39]. Secondly, dysbiosis in FC patients was reported in some studies which investigated microbiota in fecal or mucosal samples [15, 40–42]. Furthermore, germ-free mice developed constipation after receiving fecal microbiota from patients with constipation [18, 43].

## 3. Alterations of Gut Microbiota in FC

Patients with DD fail to coordinate the abdominal, rectoanal, and pelvic floor muscles during attempted defecation and are more appropriately treated with pelvic floor biofeedback therapy, indicating that this type of FC may not be related to gut microbiota [44]. Inversely, FMT has suggested that STC or NTC was associated with gut microbiota [38, 39].

Earlier studies using culture-based approaches reported that *Bifidobacterium* and *Lactobacillus* were significantly lower and potentially pathogenic bacteria or fungi were increased in adult FC patients, whereas *Bifidobacterium* and *Clostridium* were significantly higher in children patients [42, 45]. However, conventional detective techniques underestimated the diversity of the colonic microbiota by a factor of as much as 50%, which limited our further understanding of gut microbiota in patients with constipation [36]. Recently, culture-independent technologies, such as quantitative PCR using 16S rRNA gene-specific primers and high-throughput sequencing techniques, could provide insightful knowledge of the composition or the functional capacity of gut microbiota based on the detection of specific metabolic activities expressed by bacterial species, which have been widely used in most researches. For example, studies have reported that patients with constipation exhibited increased *Bacteroidetes* in colonic mucosa, while *Bacteroides* and *Bifidobacterium* were significantly decreased in feces [40, 46]. In addition, several families and genera in *Firmicutes* and butyrate-producing genera *Coprococcus*, *Roseburia*, and *Faecalibacterium* were more abundant in the constipated patients [41]. Mancabelli and colleagues unveiled functionalities of the gut microbiota associated with constipation through metagenomic analyses and reported that the microbiomes corresponding to FC exhibited high abundance of genes involved in hydrogen production, methanogenesis, and glycerol degradation [15]. Data from an animal experiment showed that the relative abundance of *Akkermansia* in the FMT-constipation group was significantly increased [43]. Moreover, Methanogens were higher in patients with STC compared to NTC or controls, which may influence gut functions by slowing colonic transit [47].

Taken together, although the findings are inconsistent to some extent and currently no general consensus and a specific microbial signature exist, the main characteristics of gut microbiota in FC patients are the relative decrease of beneficial bacteria such as *Lactobacillus* and *Bifidobacterium*, the relative increase of potential pathogens, and the reduced species richness [48]. Consequently, structural changes of gut microbiota may further contribute to dysfunction.

On the other hand, the discordances may result from different characteristics of patients and approaches used for microbiota analysis, source of samples, and different study designs. Age, diet, growth environment, living habits, personality emotions, congenital inheritance, medication factors, and anatomical structures can all affect the composition and function of gut microbiota to varying degrees, which accordingly predispose some people to different diseases, including FC [22, 25, 48–50]. For instance, a diet high in plant-derived carbohydrates has been correlated with the *Prevotella* enterotype, whereas protein and fat intake associated with the *Bacteroides* enterotype [51]. Also, the low-fiber diet is possibly a cause of microbial dysbiosis [41]. Another study pointed that the mucosal microbiota was linked to constipation, independent of colonic transit, whereas the fecal microbiota was linked to colonic transit and breath methane production [40]. Moreover, a recent study using humanized mice demonstrated that altered gut motility changed the composition of the gut microbiota, while another study proposed that transit time might shape gut microbiota richness and composition, enterotypes, and bacterial growth rates [52, 53], all suggesting that FC per se could affect the composition of gut microbiota and related functions of metabolites and that dysbiosis interacted with the dysmotility in FC in consideration of the effect of brain-gut-microbiome axis. In total, evidence for whether these alterations in gut microbiota might be either a cause or a consequence of FC remains sparse and much effort is currently concentrated on elucidating the mechanisms of the gut microbiota in the pathophysiology of this condition, such as producing some available physiologically active substances as described below.

## 4. Possible Roles of Microbial Metabolites in FC

FC patients carry gut dysfunction more or less, while gut microbiota can modulate gut functions either directly or indirectly through the metabolites of bacterial fermentation, mediators released by the gut immune response, or intestinal neuroendocrine factors [35, 54]. Here, we focus on the part which gut metabolites play in the pathophysiology of FC. However, most of our knowledge regarding possible metabolites associated with FC derives from a small amount of studies mainly in animals, among which SCFAs, bile acids, and methane are the most common metabolites.

### 4.1. Short-Chain Fatty Acids (SCFAs)

SCFAs, such as acetate, propionate, and butyrate, are mainly generated by colonic anaerobic bacterial fermentation of dietary compounds (i.e., dietary-resistant starch and nonstarch polysaccharides which are not completely digested in the small intestine) in almost constant molar ratio of 65 : 20 : 15 [55, 56]. They involve inthe host's regulation of physiological functions, including lipid or glucose de novo synthesis, host-microbe signaling, stimulating colonic blood flow, fluid and electrolyte uptake, and modulating gut motility [23, 57]. Due to their uptake and metabolism by colonocytes, especially butyrate, SCFAs have been primarily associated with colonic function [58]. Butyrate can regulate colonic mucosa homeostasis as well as neuronal excitability and may increase the cholinergic-mediated colonic circular muscle contractile response ex vivo [59].

A study predicted that increased butyrate production might contribute to constipation through reporting significantly decreased representation of *Prevotella* species which did not produce butyric acid and increased butyrate-producing genera *Coprococcus*, *Roseburia*, and *Faecalibacterium* in FC patients [41]. In another study, interestingly, butyrate concentrations were decreased significantly in mice treated with FMT from STC donors and, after supplementation with butyrate, some constipation-related symptoms in mice were reversed [18]. Wang et al. reported that acetic acid and butyric acid could relieve constipation through different ways [60]. Actually, the opposite results over the effects of SCFAs on colonic motility have been existing. For example, inhibitory effects have been shown in the large intestine of sheep and rat isolated colon [61–64]. Jouët et al. reported that infusions of a SCFA mixture did not modify the colonic motor activity in healthy volunteers, whereas another study indicated that high SCFA concentrations could lead to diarrhea [63, 64]. Evidence from animal studies has suggested that this controversy could be attributed to the concentration, the chemical nature and dose of SCFAs, responsiveness of the colonic segments, and animal species [55, 61, 62, 63]. In particular, butyrate has biphasic effects on colonic motility: it promotes motility at low concentrations, while it inhibits motility at excessive concentrations through disturbing water and electrolyte absorption and preventing mucin secretion from intestinal goblet cells [40, 41, 65]. Similarly, there were also different studies which reported that acetate and propionate enhanced gut motility or inhibited it [66, 67]. Taken together, the effect of SCFAs on colonic motility may be the net effect of these opposing influences, indicating that it is necessary to explore the role of individual SCFAs in FC.

**Figure 2:**
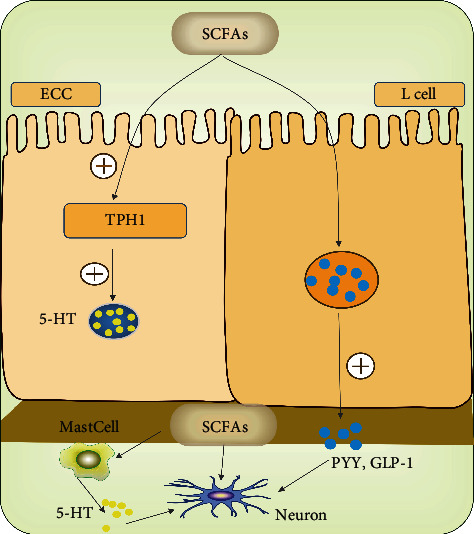
SCFAs and gut motility. SCFAs can promote colonic enzyme tryptophan hydroxylase-1 (TPH1) expression and 5-HT production by acting on EC cells and stimulate L cells to release PYY and GLP-1 as well as mucosal mast cells to release 5-HT, which then affect gut motility indirectly. Moreover, SCFAs derived from the blood can directly activate somas of myenteric intrinsic primary afferent neurons (IPANs) to regulate motility.

There are several possible underlying mechanisms of SCFAs in gut motility and intestinal transit (Figure 2). On the one hand, SCFAs can enhance the peristalsis and contraction of intestinal smooth muscle by reducing intestinal pH. In addition, low intestinal pH promotes the development of certain beneficial microbiota, particularly *Lactobacillus* and *Bifidobacterium* [65, 68]. On the other hand, SCFAs can promote colonic enzyme tryptophan hydroxylase-1 (TPH1) expression and 5-HT production by acting on ECCs [69]. 5-HT released from 5-HT-containing mucosal mast cells in response to SCFAs may induce excitatory and inhibitory physiological effects on colonic motility [70]. SCFAs can also stimulate L cells (one of the EECs) located at the distal ileum to secrete peptide YY (PYY) and glucagon-like peptide-1 (GLP-1) [71, 72]. PYY plays an inhibitory role in gut motility, while GLP-1 has a definite inhibitory effect on stomach and small intestine but the effect on colon is unclear [73, 74]. Moreover, SCFAs are not only in the colonic lumen but also in the blood, which makes it possible that SCFAs derived from the blood directly activate somas of myenteric intrinsic primary afferent neurons (IPANs) to regulate motility [66]. On the other hand, the abovementioned pathways are mediated mainly via fatty acid receptors, FFA2 and/or FFA3 [72, 75]. Nevertheless, these mechanisms have not been verified clearly in FC patients.

### 4.2. Bile Acids (BAs)

Primary BAs, including chenodeoxycholic acid (CDCA) and cholic acid (CA), are endogenous molecules synthesized from cholesterol in the liver and then enter the intestine through the bile duct, 5 to 10% of which are subject to extensive biotransformation through degradation by intestinal bacteria, mainly including anaerobic bacteria of the genera *Bacteroides*, *Eubacterium*, and *Clostridium* [17, 19, 23, 76]. These free BAs then form secondary BAs such as lithocholic acid (LCA) and deoxycholic acid (DCA), which are implicated in many physical functions via binding G protein-coupled bile acid receptor (TGR5) or farnesoid X receptor (FXR), such as bile acid synthesis and transport as well as lipid and glucose metabolism [23, 76, 77].

In addition, several studies have reported that BAs played an important part in colonic motility and secretion. There were positive correlations between colonic transit and total fecal BAs as well as percentage of fecal CDCA and a negative correlation was observed between colonic transit and percentage of fecal LCA [19]. This study also found that CDCA and DCA, potent secretory BAs, decreased in feces of IBS-C, whereas nonsecretory LCA increased. Actually, the prosecretory effects of BAs have been considered as structure-specific and dependent on bacterial actions and both prosecretory and nonsecretory BAs can convert to each other [76, 78]. Altered BA synthesis also showed a significant correlation with colonic transit in FC patients; that is, 92% of the patients with NTC had an increase in C4 (a marker of de novo hepatic BA synthesis) at lunchtime, while 82% of the patients with STC had no increase in C4. However, the higher concentration of C4 in the morning was observed in patients with severe delayed colonic transit. This study indicated that patients with constipation might have a disturbed diurnal rhythm of the BA synthesis [79]. Moreover, sulfation of CDCA may be related to constipation and 3-sulfate of CDCA should not be involved in the enterohepatic cycling [78]. For FC patients, therefore, given that BA biosynthesis has been known to follow negative feedback regulation, this process may be increased in response to the severely delayed colonic transit time, which may be associated with BA biotransformation, such as 3-sulfate of CDCA, but the colon does not respond to the increased BAs [78, 79].

To put it in another way, decreased secretory BAs or sulfation of BAs could be the pathophysiological factors of constipation. One study in mice after receiving fecal microbiota from STC patients showed that there was a tendency towards decreased concentrations of secondary BAs, whereas no significant difference was found in primary BAs. After supplementation with DCA, some symptoms in mice from STC donors got better [18]. Altogether, future studies should emphasize the role of detection of total fecal BAs and the percentages of individual primary and secondary BAs in FC.

**Figure 3:**
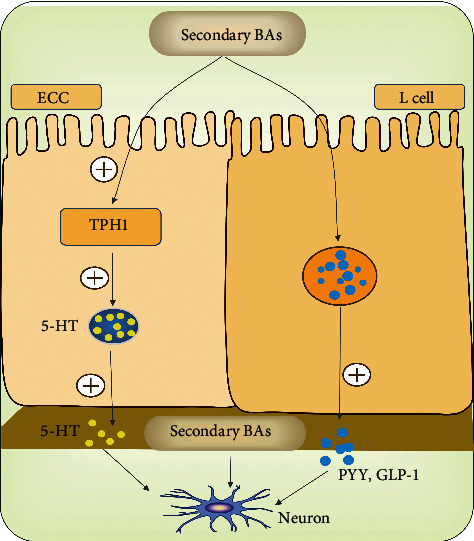
BAs and gut motility. Similar to SCFAs, BAs can stimulate L cells or ECCs to release PYY, GLP-1, and 5-HT respectively. Furthermore, BAs can directly act on IPANs via activating TGR5, which result in the inhibition of proximal intestinal transit and colonic motility.

Secondary BAs were considered to directly act on ECCs to synthetize 5-HT by upregulated TPH1 expression and to stimulate the release of 5-HT and calcitonin gene-related peptide (CGRP) from ECCs and to act on IPANs or L cells via activating G protein-coupled bile acid receptor (TGR5) (Figure 3), which resulted in the inhibition of proximal intestinal transit and colonic motility [77, 80, 81]. In contrast, CDCA can accelerate colonic transit and enhance high amplitude propagated contractions probably via a different manner, which may result from a higher concentration of the intraluminal BAs than the dose that activates the neural or hormonal mechanisms [77]. In summary, it is probable that BAs involve in the development of constipation by modulating the release of 5-HT from ECCs or activating TGR5 located in the colon.

### 4.3. Methane

In humans, methane is generated mainly by methanogens in colon using CO_2_ and H_2_ which is produced through anaerobic fermentation of undigested polysaccharide fraction [82]. Methane is excreted either in flatus or in breath, approximately 20%–50% of which is thought to be excreted through lungs; hence, lactulose breath test (LBT) can indirectly measure their production [20]. Evidence from clinical and animal experiments is accumulating to suggest that methane is associated with intestinal transit time. Studies from children and adults with chronic constipation have demonstrated that colonic transit time was significantly prolonged in methane producers [47, 83]. Prevalence of methanogens and methane production were both higher in the STC group than in the NTC group [47]. Furthermore, constipation severity was significantly related to the quantity of methane production during the LBT, whereas methane production was correlated to a lower number of bowel movements in IBS patients and antibiotic treatment could improve symptoms of constipation due to elimination of methane in some patients [20, 84]. In animal models, methane has been seen to increase nonpropagating small bowel contractile activity and decrease small bowel transit in an ex vivo guinea pig ileum experiment, while methane produced a slower transit by an average of 59% compared to room air in canine models [85]. Taken together, methane is related to FC, especially to STC.

Considering that methane involves in gastrointestinal dysmotility to some extent, it may not be an inert intestinal gas; in contrast, it is a bioactive molecule which can act like a neuromuscular transmitter to influence the gut motility and contribute to FC [20]. A study on IBS reported that 4 of 18 patients produced methane, and postprandial 5-HT concentration was decreased compared to the H2-producing patients, while another study regarding the anesthesia reported that halogenated methane was able to inhibit the pulmonary uptake of 5-HT in rat lung and the degree of chlorination was the most important factor that affected the inhibitory effect [86, 87]. Based on the above data, we make assumptions that methane can participate in the pathogenesis of constipation via regulating 5-HT concentration, just like SCFAs and BAs. However, to date, it is not clear whether these two molecules really work together to influence gut motility.

On the contrary, a study pointed that methane production was not associated with constipation or colonic transit [40]. Actually, methane is detected in a certain proportion of healthy people and cannot account for all FC cases [20]. Moreover, an absence of methane in the LBT does not indicate absence of methanogens. Therefore, methane is not the only cause of constipation.

### 4.4. Other Possible Microbial Metabolites

Except the above-described metabolites, reduced neurotransmitter concentrations (such as nitric oxide and vasoactive intestinal peptide) were reported in some patients with STC [7, 18, 88]. Other fermentation byproducts, such as alcohols, ketones, and aldehydes, may also influence gut motility [35]. Recently, a study in neonatal maternal separation (NMS) rats reported that an excess of saturated long-chain fatty acid (SLCFAs), positively correlated with elevated abundances of *Prevotella*, *Lactobacillus*, and *Alistipes*, induced enhanced bowel motility, which suggested the importance of SLCFA-producing bacteria in GI motility disorders [89].

Taken together, the gut microbiota interacts with the host to produce plenty of biologically active molecules during the metabolism of food, which indeed involves in the pathological processes of FC in a variety of ways when there is a dysbiosis. However, current findings are still controversial, such as the precise effects of individual SCFAs in FC patients. Pathogenic mechanisms of some metabolites have not been clear yet, such as methane and SLCFAs.

## 5. 5-Hydroxytryptamine (5-HT)

5-HT, also called serotonin, is not only an important neurotransmitter but also an important regulatory factor in the gastrointestinal tract mainly (90%) synthesized by ECCs using amino acid tryptophan (Trp) and TpH-1 [81]. Luminal chemical and mechanical stimuli activate corresponding receptors distributed in ECCs or mucosal mast cells to release 5-HT into the interstitial space of the lamina propria, intestinal lumen, or the blood, which further participate in regulating gut motility and secretory, vasodilatory reflexes as well as sending signals to the brain and spinal cord via binding corresponding receptors [90, 91]. Serotonin transporter (SERT), a transmembrane transport protein predominantly expressed by all epithelial cells of the gut mucosa, reuptakes excessive 5-HT to terminate its physiological effects, that is, regulates extracellular 5-HT availability [43].

**Figure 4:**
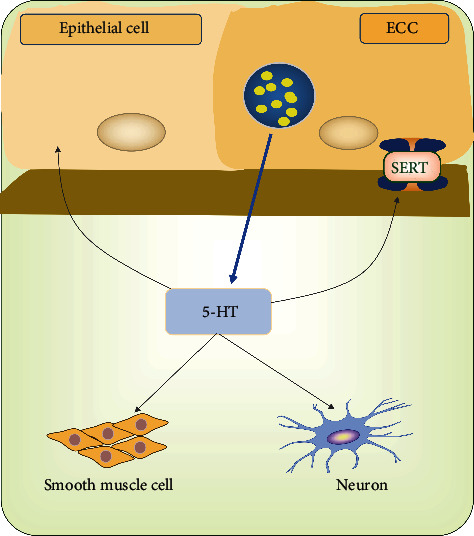
5-HT and gut motility. 5-HT, released from ECCs or mucosal mast cells, can contribute to smooth muscle contraction or relaxation and regulate gut sensation and secretion via various receptors located on smooth muscle cells, enteric neurons, and epithelial cells, mainly including 5-HT1, 2, 3, 4, and 7 subtypes. On the other hand, serotonin transporter (SERT), predominantly expressed by all gut epithelial cells, reuptakes excessive 5-HT to terminate its physiological effects.

Increasing evidence also has indicated that 5-HT regulated gut motility as downstream signaling molecules of metabolites derived from microbiota and alterations in 5-HT signaling might contribute to FC (Figure 4). A recent study has demonstrated that indigenous spore-forming microbes from the gut microbiota produced metabolites that promoted host 5-HT biosynthesis in the gastrointestinal tract and impacted gut motility [81]. On the one hand, studies regarding gut microbiota in FC patients reported that microbial metabolites might induce constipation through regulating a significant percentage of 5-HT synthesis and release, such as SCFAs and BAs as mentioned above. In fact, due to multiple receptor subtypes, 5-HT has complex biological activities. In terms of gut function, 5-HT can result in smooth muscle contraction or relaxation and regulate gut sensation and secretion via various receptors located on smooth muscle cells, enteric neurons, and epithelial cells, mainly including 5-HT1, 2, 3, 4, and 7 subtypes [92]. For example, neuronal 5-HT receptors, including the 5-HT1A (inhibitory) [93], 5-HT3, and 5-HT4 (both excitatory) subtypes [94], may inhibit or enhance transmitter release. Therefore, the combined effects of activation of these receptors may be indicative of the ultimate phenotype. On the other hand, several studies on animals and human with constipation explored the concentrations of SERT and 5-HT in colonic tissue but got different results and conclusions. An animal study indicated that gut dysbiosis might lead to the development of CC via upregulating the expression of SERT and then decreasing 5-HT concentration, which could weaken the intestinal circular muscle contraction activity and inhibit gut motility [43]. But other studies reported that increased 5-HT concentrations of the blood or the colonic mucosa were related to constipation. For example, one reported that increased PDP 5-HT concentration associated with visceral insensitivity and reduced stool frequency rather than colonic transit in patients with constipation [88]. Another proposed that increased 5-HT availability, which resulted from increased synthesis and release other than decreased SERT expression, might contribute to constipation due to receptor desensitization [90]. From a different perspective, studies on guinea pigs have reported that low-dose fluoxetine (i.e., one of the selective serotonin reuptake inhibitors) enhanced intestinal propulsion, while higher doses slowed or blocked propulsion, indicating again the potential importance of available 5-HT and a possible balance between receptor activation and desensitization in the outcome on gastrointestinal motility and transit [88, 95]. However, patients with IBS-D exhibited lower SERT levels and high 5-HT concentrations in the colon mucosa [96]. All in all, it is really tricky to explain these paradoxical findings. But, intriguingly, Guarino M et al. resolved the conflict to some extent through in vivo and in vitro experiments [21]. They demonstrated that overexpressed progesterone (P4) receptors in females with STC induced low SERT and high 5-HT levels as well as normal TPH-1. Moreover, increases in 5-HT, which were ineffective because overexpression of P4 receptors in muscle cells impaired the contraction of the circular muscle layer in response to 5-HT and ACh, might represent a compensatory mechanism to increased transit time in female patients with STC. However, regardless of receptor desensitization or compensatory mechanism, the elevated 5-HT levels in constipation patients deserve further study.

## 6. Microbial-Associated Treatments and Research Strategies in FC

Given the potential effects of gut microbiota and metabolites on the occurrence and development of FC, there is an increasing interest in the corresponding targeted therapies, such as some probiotics, prebiotics, and synbiotics, ileal bile acid transporter inhibitors, antibiotic therapy in methanogenic patients, and serotonin 5-HT4 receptor agonists, which have achieved efficacy to some extent [20, 34, 35, 44]. Although high-quality but limited clinic data on probiotics in constipation focused on the improvement of constipation-related symptoms, such as defecation frequency or stool consistency, microbiota-related preparations also have a vital role in the regulation of host immune system [36, 97–99]. For example, probiotic bacteria, especially *Lactobacilli* and *Bifidobacteria*, can stimulate immune cells (e.g., Th1, Th2, Th17, T regulator cells, and B cells) and the release of antimicrobial substances (e.g., mucin),increase sIgA production and the formation of macrophages to defend the pathogenic bacteria and toxins in the gut, and thus maintain intestinal barrier integrity [99–102]. However, the safety of probiotics has been questioned in recent studies, particularly in older patients with an impaired intestinal mucosal barrier or immunosuppressive state, which might cause microbial translocation, infections of opportunistic pathogens, D-lactic acidosis, and loss of bioactivity of antibacterial or antifungal drugs [103–105]. Fortunately, lines of evidence indicated that the preparation of nonviable microorganisms and/or components produced from probiotics, called postbiotics, showed similar health benefits to probiotics while eliminating the abovementioned safety problems [99, 106, 107]. Butyrate, a bacterial metabolite, also belongs to postbiotics, which can regulate macrophage function through the inhibition of histone deacetylases and thus renders the intestinal immune system hyporesponsive to the commensal bacteria and exhibits anti-inflammatory effects [108]. Although we have realized that butyrate could influence colonic motility of constipation patients as described above, how it regulates intestinal immune responses in FC remains unknown. Indeed, few studies regarding immune system manifestations of FC have been conducted, despite the fact that the immune system influences gut motility [42, 109, 110]. Thus, gut immunity in FC needs further research and may become a new therapeutic target.

In addition to the above common drugs, botanical laxatives or herbal medicines, including Chinese medicine (CM), have been used to relieve constipation, especially in East Asia [25]. Anthraquinone drugs (e.g., senna, aloe, rhubarb, frangula, and cascara) are the most commonly used botanical laxatives, mainly by stimulating fluid secretion and improving altered motility patterns to facilitate constipation, especially applicable for short-term treatment of atonic constipation, acute constipation, and before endoscopy of the lower gastrointestinal tract [111–113]. However, due to the side effects from long-term application [112], anthraquinone drugs are not recommended as the first choice for constipation clinically. The genus *Gynura* was also reported to treat constipation [114]. Except single herbal medicines, many herbal formulas also alleviate constipation. Xiao'er Biantong Granules, Huangxin Runchang Pian, and Modified Buzhong-Yiqi-Tang significantly improved the constipation symptoms (e.g., frequencies of spontaneous bowel movements, stool consistency, excessive straining, or a sense of incomplete evacuation), while no severe adverse effects were observed [115–117]. Intriguingly, CM formulas have been proven to deal with diseases by restoring the normal composition and function of gut microbiota and regulating metabolites in clinical or animal experimental studies, including constipation [118–121]. For example, Zengye Decoction (ZYD), consisting of Radix Scrophulariae, *Ophiopogon japonicus*, and Radix Rehmannia, reduced the abundance of harmful microbes (e.g., *Desulfovibrio*, *Prevotella*, and *Ruminococcus*), while it increased the abundance of *Oxalobacter*, *Clostridium*, and *Roseburia* in elderly constipation rats [122]. Tong Bian Decoction increased SCFAs and butyric acid in feces of senile constipation [123]. Xu et al. pointed that gut microbiota also conformed to CM holistic concept, CM Yin-Yang balance theory, and CM constitutional theory [25]. This explains the efficacy of CM formulas in the management of constipation and indicates that supplement of a single strain may not achieve the desired effect and individualized therapy is essential. However, some CM formulas usually functioned through multitarget and multipath mechanisms and the microbiota-related mechanisms of action have not been studied extensively [124]. In addition, CM is the precious treasure of China, which deserves further research. In short, in-depth and extensive exploration of the pathological mechanisms of FC will dig out more targeted treatments and benefit more patients.

In the past few decades, microbiological researches were paid much attention and achieved great results with the implementation of the Genome Project as well as development of new-generation sequencing technologies. People have come to recognize that alterations in microbial composition contributed to human diseases. However, in fact, these microbial detection techniques only focused on differences in composition, which cannot further explore the functional level of the differential microbiota, so as to better elucidate the pathogenesis of diseases and develop new therapeutic drugs. Recently, the development of multiomics techniques addressed the above problems. For example, metagenomics can identify novel functional genes, microbial pathways, antibiotic resistance genes, and functional dysbiosis of the gut microbiome and determine interactions and coevolution between microbiota and host [125]; metabolomics can reveal and confirm new pathways and identify novel metabolic biomarkers among different physiological conditions by obtaining metabolic profiles [126]. Furthermore, metatranscriptomics and metaproteomics also provide enormous complements to the understanding of the human gut microbiome [127, 128]. The serum metabolite, 1-methyladenosine, was identified as a characteristic metabolite for hepatocarcinoma (HCC) [129]. A recent study identified 122 robust associations between differentially abundant species and metabolites and reported that metabolome- and metagenome-based classifiers of IBD status were accurate, which provided an improved understanding of perturbations of the microbiome-metabolome interface in IBD, including identification of many potential diagnostic and therapeutic targets [130].

In contrast, current studies on FC tended to consider one aspect of microbial mechanisms, that is, either microbial aspects or metabolic profiles, which may get one-sided conclusions to a certain extent. If applying multiomics techniques to studies (e.g., combination of metagenomics and metabolomics), we perhaps have a more complete understanding of FC, including structural, functional, and metabolic levels, and can identify more precise and specific gut microbes or metabolites as targets for therapeutic and preventive interventions. In addition, animals are common subjects in most FC studies (i.e., preclinical studies), and findings from animals may be inconsistent with those from human sometimes. For example, the microbial metabolism of BAs is different between mice and humans, which may result in different signaling pathways involving in the pathogenesis of FC [131]. This translational inconsistency highlights the possibility of host-specific microbiota interactions and emphasizes the importance of cautious extrapolation of preclinical findings as well as high-quality clinical trials. Of course, in order to get more scientific and reasonable conclusions, future study designs should stratify FC and take age, gender, diet, region, race, or nationality into account.

## 7. Conclusions

Functional constipation (FC) is one of the most common functional gastrointestinal disorders (FGIDs) in the world, which influences the quality of life and results in a high economic burden on the healthcare services. Previous studies have reported that colonic sensorimotor dysfunction and pelvic floor dysfunction were the main pathogenesis, which classified FC into four subgroups: normal transit constipation (NTC), slow transit constipation (STC), defecatory disorders (DD), and mixed type. Recently, with the proposition of the concept of the brain-gut-microbiome axis and its association with many diseases, the role of gut microbiota in the pathophysiology of FC has drawn more attention. Studies have reported that microbial dysbiosis occurred in FC patients and metabolites derived from interaction of the host and gut microbiota, as an intermediate link, contributed to the development of this disorder via various signal pathways, among which SCFAs, BAs, and methane occupied a more important position. 5-HT, as an essential neurotransmitter and regulatory factor of the gastrointestinal tract, also involved in the modulation of gut motility, secretory as well as sensory transmission in patients with constipation. Altogether, current studies have provided us a new conception on the microbial mechanisms and therapeutic targets of constipation. However, research findings were inconsistent and even conflicting, which may be partially attributed to many confounding factors, such as age, diets, source of samples, and study subjects. Meanwhile, gut microbiota is only a tip of the iceberg in the pathophysiology of FC. Therefore, future studies should control for the associated confounders and adopt multiomics research strategies (e.g., metagenomics, metabolomics, metatranscriptomics, and metaproteomics) to conduct microbial researches of FC, which contribute to obtaining more complete and in-depth conclusions as well as providing reliable theoretical support for exploring new therapeutic targets.

## References

[1] Bharucha A. E., Pemberton J. H., Locke G. R. (2013). American gastroenterological association technical review on constipation. *Gastroenterology*.

[2] Forootan M., Bagheri N., Darvishi M. (2018). Chronic constipation: a review of literature. *Medicine*.

[3] Schmidt F. M. Q., De Gouveia Santos V. L. C. (2014). Prevalence of constipation in the general adult population: an integrative review. *Journal of Wound, Ostomy & Continence Nursing*.

[4] Rajindrajith S., Devanarayana N. M., Crispus Perera B. J., Benninga M. A. (2016). Childhood constipation as an emerging public health problem. *World Journal of Gastroenterology*.

[5] Sharma A., Rao S. (2017). Constipation: pathophysiology and current therapeutic approaches. *Gastrointestinal Pharmacology*.

[6] Moezi P., Salehi A., Molavi H. (2018). Prevalence of chronic constipation and its associated factors in pars cohort study: a study of 9000 adults in southern Iran. *Middle East Journal of Digestive Diseases*.

[7] Lacy B. E., Mearin F., Chang L. (2016). Bowel disorders. *Gastroenterology*.

[8] Serra J., Pohl D., Azpiroz F. (2020). European society of neurogastroenterology and motility guidelines on functional constipation in adults. *Neuro-Gastroenterology and Motility: The Official Journal of the European Gastrointestinal Motility Society*.

[9] Huang L., Jiang H., Zhu M. (2017). Prevalence and risk factors of chronic constipation among women aged 50 years and older in Shanghai, China. *Medical Science Monitor*.

[10] Bharucha A. E., Wald A. (2019). Chronic constipation. *Mayo Clinic Proceedings*.

[11] Bharucha A. E., Lacy B. E. (2020). Mechanisms, evaluation, and management of chronic constipation. *Gastroenterology*.

[12] Vriesman M. H., Koppen I. J. N., Camilleri M., Di Lorenzo C., Benninga M. A. (2020). Management of functional constipation in children and adults. *Nature Reviews Gastroenterology & Hepatology*.

[13] Wedel T., Spiegler J., Soellner S. (2002). Enteric nerves and interstitial cells of cajal are altered in patients with slow-transit constipation and megacolon. *Gastroenterology*.

[14] Simrén M., Barbara G., Flint H. J. (2013). Intestinal microbiota in functional bowel disorders: a Rome foundation report. *Gut*.

[15] Mancabelli L., Milani C., Lugli G. A. (2017). Unveiling the gut microbiota composition and functionality associated with constipation through metagenomic analyses. *Scientific Reports*.

[16] Hills R. D., Pontefract B. A., Mishcon H. R., Black C. A., Sutton S. C., Theberge C. R. (2019). Gut microbiome: profound implications for diet and disease. *Nutrients*.

[17] Martin C. R., Osadchiy V., Kalani A., Mayer E. A. (2018). The brain-gut-microbiome axis. *Cellular and Molecular Gastroenterology and Hepatology*.

[18] Ge X., Zhao W., DIng C. (2017). Potential role of fecal microbiota from patients with slow transit constipation in the regulation of gastrointestinal motility. *Scientific Reports*.

[19] Shin A., Camilleri M., Vijayvargiya P. (2013). Bowel functions, fecal unconjugated primary and secondary bile acids, and colonic transit in patients with irritable bowel syndrome. *Clinical Gastroenterology and Hepatology*.

[20] Triantafyllou K., Chang C., Pimentel M. (2014). Methanogens, methane and gastrointestinal motility. *Journal of Neurogastroenterology and Motility*.

[21] Guarino M., Cheng L., Cicala M., Ripetti V., Biancani P., Behar J. (2011). Progesterone receptors and serotonin levels in colon epithelial cells from females with slow transit constipation. *Neuro-Gastroenterology and Motility*.

[22] Lynch S. V., Pedersen O. (2016). The human intestinal microbiome in health and disease. *New England Journal of Medicine*.

[23] Nicholson J. K., Holmes E., Kinross J. (2012). Host-gut microbiota metabolic interactions. *Science*.

[24] Tremaroli V., Bäckhed F. (2012). Functional interactions between the gut microbiota and host metabolism. *Nature*.

[25] Xu Z., Liu T., Zhou Q., Chen J., Yuan J., Yang Z. (2019). Roles of Chinese medicine and gut microbiota in chronic constipation. *Evidence-Based Complementary and Alternative Medicine*.

[26] Burcelin R., Courtney M., Amar J. (2015). Gut microbiota and metabolic diseases: from pathogenesis to therapeutic perspective. *Molecular and Integrative Toxicology*.

[27] Kamo T., Akazawa H., Suda W. (2017). Dysbiosis and compositional alterations with aging in the gut microbiota of patients with heart failure. *PLoS One*.

[28] Loomba R., Seguritan V., Li W. (2017). Gut microbiome-based metagenomic signature for non-invasive detection of advanced fibrosis in human nonalcoholic fatty liver disease. *Cell Metabolism*.

[29] Ranasinghe N., Devanarayana N. M., Benninga M. A., van Dijk M., Rajindrajith S. (2017). Psychological maladjustment and quality of life in adolescents with constipation. *Archives of Disease in Childhood*.

[30] Philips E. M., Peeters B., Teeuw A. H. (2015). Stressful life events in children with functional defecation disorders. *Journal of Pediatric Gastroenterology and Nutrition*.

[31] Haug T. T., Mykletun A., Dahl A. A. (2002). Are anxiety and depression related to gastrointestinal symptoms in the general population?. *Scandinavian Journal of Gastroenterology*.

[32] Rajindrajith S., Devanarayana N. M., Benninga M. A. (2010). Constipation-associated and nonretentive fecal incontinence in children and adolescents: an epidemiological survey in Sri Lanka. *Journal of Pediatric Gastroenterology and Nutrition*.

[33] Mugie S. M., Koppen I. J. N., van den Berg M. M. (2018). Brain processing of rectal sensation in adolescents with functional defecation disorders and healthy controls. *Neuro-Gastroenterology and Motility*.

[34] Shatnawi M., Alrwala M., Ghanma A., Alquraan M., Zreiqat E., Alzoubi M. (2019). Lactulose versus polyethylene glycol for disimpaction therapy in constipated children, a randomized controlled study. *Sudanese Journal of Paediatrics*.

[35] Choi C. H., Chang S. K. (2015). Alteration of gut microbiota and efficacy of probiotics in functional constipation. *Journal of Neurogastroenterology and Motility*.

[36] Quigley E. M. M. (2011). The enteric microbiota in the pathogenesis and management of constipation. *Best Practice & Research Clinical Gastroenterology*.

[37] Ge X., Tian H., Ding C. (2016). Fecal microbiota transplantation in combination with soluble dietary fiber for treatment of slow transit constipation: a pilot study. *Archives of Medical Research*.

[38] Tian H., Ding C., Gong J. (2016). Treatment of slow transit constipation with fecal microbiota transplantation. *Journal of Clinical Gastroenterology*.

[39] Ohara T. (2019). Identification of the microbial diversity after fecal microbiota transplantation therapy for chronic intractable constipation using 16s rRNA amplicon sequencing. *PLoS One*.

[40] Parthasarathy G., Chen J., Chen X. (2016). Relationship between microbiota of the colonic mucosa vs feces and symptoms, colonic transit, and methane production in female patients with chronic constipation. *Gastroenterology*.

[41] Zhu L., Liu W., Alkhouri R. (2014). Structural changes in the gut microbiome of constipated patients. *Physiological Genomics*.

[42] Khalif I., Quigley E., Konovitch E., Maximova I. (2005). Alterations in the colonic flora and intestinal permeability and evidence of immune activation in chronic constipation. *Digestive and Liver Disease*.

[43] Cao H., Liu X., An Y. (2017). Dysbiosis contributes to chronic constipation development via regulation of serotonin transporter in the intestine. *Scientific Reports*.

[44] Prichard D. O., Bharucha A. E. (2018). Recent advances in understanding and managing chronic constipation. *F1000Research*.

[45] Zoppi G., Cinquetti M., Luciano A., Benini A., Muner A., Bertazzoni Minelli E. (1998). The intestinal ecosystem in chronic functional constipation. *Acta Paediatrica (Oslo, Norway: 1992)*.

[46] Kim S.-E., Choi S. C., Park K. S. (2015). Change of fecal flora and effectiveness of the short-term VSL#3 probiotic treatment in patients with functional constipation. *Journal of Neurogastroenterology and Motility*.

[47] Attaluri A., Jackson M., Valestin J., Rao S. S. (2010). Methanogenic flora is associated with altered colonic transit but not stool characteristics in constipation without IBS. *American Journal of Gastroenterology*.

[48] Ohkusa T., Koido S., Nishikawa Y., Sato N. (2019). Gut microbiota and chronic constipation: a review and update. *Frontiers of Medicine*.

[49] Zhao Y., Yu Y. B. (2016). Intestinal microbiota and chronic constipation. *SpringerPlus*.

[50] Rajilić-Stojanović M., Jonkers D. M., Salonen A. (2015). Intestinal microbiota and diet in IBS: causes, consequences, or epiphenomena?. *American Journal of Gastroenterology*.

[51] Wu G. D., Chen J., Hoffmann C. (2011). Linking long-term dietary patterns with gut microbial enterotypes. *Science*.

[52] Kashyap P. C., Marcobal A., Ursell L. K. (2013). Complex interactions among diet, gastrointestinal transit, and gut microbiota in humanized mice. *Gastroenterology*.

[53] Vandeputte D., Falony G., Vieira-Silva S., Tito R. Y., Joossens M., Raes J. (2016). Stool consistency is strongly associated with gut microbiota richness and composition, enterotypes and bacterial growth rates. *Gut*.

[54] Barbara G., Stanghellini V., Brandi G. (2005). Interactions between commensal bacteria and gut sensorimotor function in health and disease. *American Journal of Gastroenterology*.

[55] Dass N. B., John A. K., Bassil A. K. (2007). The relationship between the effects of short-chain fatty acids on intestinal motility in vitro and GPR43 receptor activation. *Neuro-Gastroenterology and Motility*.

[56] Fukumoto S., Tatewaki M., Yamada T. (2003). Short-chain fatty acids stimulate colonic transit via intraluminal 5-HT release in rats. *American Journal of Physiology. Regulatory, Integrative and Comparative Physiology*.

[57] Schwiertz A., Taras D., Schäfer K. (2010). Microbiota and SCFA in lean and overweight healthy subjects. *Obesity*.

[58] Wong J. M., Jenkins D. J. (2007). Carbohydrate digestibility and metabolic effects. *Journal of Nutrition*.

[59] Canani R. B., Costanzo M., Leone L., Pedata M., Meli R., Calignano A. (2011). Potential beneficial effects of butyrate in intestinal and extraintestinal diseases. *World Journal of Gastroenterology*.

[60] Wang L., Cen S., Wang G. (2020). Acetic acid and butyric acid released in large intestine play different roles in the alleviation of constipation. *Journal of Functional Foods*.

[61] Svendsen P. (1972). Inhibition of cecal motility in sheep by volatile fatty acids. *Nordisk Veterinaermedicin*.

[62] Yajima T. (1985). Contractile effect of short-chain fatty acids on the isolated colon of the rat. *The Journal of Physiology*.

[63] Jouët P., Moussata D., Duboc H. (2013). Effect of short-chain fatty acids and acidification on the phasic and tonic motor activity of the human colon. *Neuro-Gastroenterology and Motility*.

[64] Fritz E., Hammer H. F., Lipp R. W., Hogenauer C., Stauber R., Hammer J. (2005). Effects of lactulose and polyethylene glycol on colonic transit. *Alimentary Pharmacology and Therapeutics*.

[65] Kwiatkowska M., Krogulska A. (2021). The significance of the gut microbiome in children with functional constipation. *Advances in Clinical and Experimental Medicine*.

[66] Mitsui R., Ono S., Karaki S., kuwahara A. (2005). Neural and non-neural mediation of propionate-induced contractile responses in the rat distal colon. *Neuro-Gastroenterology and Motility*.

[67] Hurst N. R., Kendig D. M., Murthy K. S., Grider J. R. (2014). The short chain fatty acids, butyrate and propionate, have differential effects on the motility of the Guinea pig colon. *Neuro-Gastroenterology and Motility*.

[68] de Moraes J. G., Motta M. E., Beltrão M. F., Salviano T. L., da Silva G. A. P. (2016). Fecal microbiota and diet of children with chronic constipation. *International Journal of Pediatrics*.

[69] Reigstad C. S., Salmonson C. E., Iii J. F. R. (2015). Gut microbes promote colonic serotonin production through an effect of short‐chain fatty acids on enterochromaffin cells. *The FASEB Journal*.

[70] Karaki S.-I., Mitsui R., Hayashi H. (2006). Short-chain fatty acid receptor, GPR43, is expressed by enteroendocrine cells and mucosal mast cells in rat intestine. *Cell and Tissue Research*.

[71] Larraufie P., Martin-Gallausiaux C., Lapaque N. (2018). SCFAs strongly stimulate PYY production in human enteroendocrine cells. *Scientific Reports*.

[72] Kaji I., Karaki S.-I., Kuwahara A. (2014). Short-chain fatty acid receptor and its contribution to glucagon-like peptide-1 release. *Digestion*.

[73] Wang L., Gourcerol G., Yuan P. Q. (2010). Peripheral peptide YY inhibits propulsive colonic motor function through Y2 receptor in conscious mice. *American Journal of Physiology-Gastrointestinal and Liver Physiology*.

[74] Li Z.-Y., Zhang N., Wen S. (2017). Decreased glucagon-like peptide-1 correlates with abdominal pain in patients with constipation-predominant irritable bowel syndrome. *Clinics and Research in Hepatology and Gastroenterology*.

[75] Nøhr M., Pedersen M., Gille A. (2013). GPR41/FFAR3 and GPR43/FFAR2 as cosensors for short-chain fatty acids in enteroendocrine cells vs FFAR3 in enteric neurons and FFAR2 in enteric leukocytes. *Endocrinology*.

[76] Appleby R. N., Walters J. R. F. (2014). The role of bile acids in functional GI disorders. *Neuro-Gastroenterology and Motility*.

[77] Camilleri M., Vazquez-Roque M. I., Carlson P., Burton D., Wong B. S., Zinsmeister A. R. (2011). Association of bile acid receptor TGR5 variation and transit in health and lower functional gastrointestinal disorders. *Neuro-Gastroenterology and Motility*.

[78] Hofmann A. F., Loening-Baucke V., Lavine J. E. (2008). Altered bile acid metabolism in childhood functional constipation: inactivation of secretory bile acids by sulfation in a subset of patients. *Journal of Pediatric Gastroenterology and Nutrition*.

[79] Abrahamsson H., Östlund-Lindqvist A.-M., Nilsson R., Simrén M., Gillberg P.-G. (2008). Altered bile acid metabolism in patients with constipation-predominant irritable bowel syndrome and functional constipation. *Scandinavian Journal of Gastroenterology*.

[80] Bunnett N. W. (2014). Neuro-humoral signalling by bile acids and the TGR5 receptor in the gastrointestinal tract. *The Journal of Physiology*.

[81] Yano J. M., Yu K., Donaldson G. P. (2015). Indigenous bacteria from the gut microbiota regulate host serotonin biosynthesis. *Cell*.

[82] Sahakian A. B., Jee S.-R., Pimentel M. (2010). Methane and the gastrointestinal tract. *Digestive Diseases and Sciences*.

[83] Soares A. C. F., Lederman H. M., Fagundes-Neto U., de Morais M. B. (2005). Breath methane associated with slow colonic transit time in children with chronic constipation. *Journal of Clinical Gastroenterology*.

[84] Chatterjee S., Park S., Low K., Kong Y., Pimentel M. (2007). The degree of breath methane production in IBS correlates with the severity of constipation. *American Journal of Gastroenterology*.

[85] Pimentel M., Lin H. C., Enayati P. (2006). Methane, a gas produced by enteric bacteria, slows intestinal transit and augments small intestinal contractile activity. *American Journal of Physiology-Gastrointestinal and Liver Physiology*.

[86] Pimentel M., Kong Y., Park S. (2004). IBS subjects with methane on lactulose breath test have lower postprandial serotonin levels than subjects with hydrogen. *Digestive Diseases and Sciences*.

[87] Hede A. R., Andersson L., Post C. (1985). Effect of a homologous series of halogenated methanes on pulmonary uptake of 5-hydroxytryptamine in isolated perfused rat lung. *Acta Pharmacologica et Toxicologica*.

[88] Shekhar C., Monaghan P. J., Morris J. (2013). Rome III functional constipation and irritable bowel syndrome with constipation are similar disorders within a spectrum of sensitization, regulated by serotonin. *Gastroenterology*.

[89] Zhao L., Huang Y., Lu L. (2018). Saturated long-chain fatty acid-producing bacteria contribute to enhanced colonic motility in rats. *Microbiome*.

[90] Costedio M., Coates M., Brooks E. (2009). Mucosal serotonin signaling is altered in chronic constipation but not in opiate-induced constipation. *American Journal of Gastroenterology*.

[91] Gershon M. D., Tack J. (2007). The serotonin signaling system: from basic understanding to drug development for functional GI disorders. *Gastroenterology*.

[92] Sikander A., Rana S. V., Prasad K. K. (2009). Role of serotonin in gastrointestinal motility and irritable bowel syndrome. *Clinica Chimica Acta*.

[93] Dietrich C., Kilbinger H. (1996). 5-HT1A receptor-mediated inhibition of acetylcholine release from Guinea pig myenteric plexus: potential mechanisms. *Neuropharmacology*.

[94] Costedio M. M., Hyman N., Mawe G. M. (2007). Serotonin and its role in colonic function and in gastrointestinal disorders. *Diseases of the Colon & Rectum*.

[95] Wade P., Chen J., Jaffe B., Kassem I., Blakely R., Gershon M. (1996). Localization and function of a 5-HT transporter in crypt epithelia of the gastrointestinal tract. *Journal of Neuroscience*.

[96] Faure C., Patey N., Gauthier C., Brooks E. M., Mawe G. M. (2010). Serotonin signaling is altered in irritable bowel syndrome with diarrhea but not in functional dyspepsia in pediatric age patients. *Gastroenterology*.

[97] Coccorullo P., Strisciuglio C., Martinelli M., Miele E., Greco L., Staiano A. (2010). Lactobacillus reuteri (DSM 17938) in infants with functional chronic constipation: a double-blind, randomized, placebo-controlled study. *The Journal of Pediatrics*.

[98] Chmielewska A., Szajewska H. (2010). Systematic review of randomised controlled trials: probiotics for functional constipation. *World Journal of Gastroenterology*.

[99] Yeşilyurt N., Yılmaz B., Ağagündüz D., Capasso R. (2021). Involvement of probiotics and postbiotics in the immune system modulation. *Biologics*.

[100] Dargahi N., Johnson J., Donkor O., Vasiljevic T., Apostolopoulos V. (2019). Immunomodulatory effects of probiotics: can they be used to treat allergies and autoimmune diseases?. *Maturitas*.

[101] Mack D. R., Michail S., Wei S., McDougall L., Hollingsworth M. A. (1999). Probiotics inhibit enteropathogenic *E. coli* adherence in vitro by inducing intestinal mucin gene expression. *American Journal of Physiology-Gastrointestinal and Liver Physiology*.

[102] Cebra J. J. (1999). Influences of microbiota on intestinal immune system development. *American Journal of Clinical Nutrition*.

[103] Camilleri M. (2019). Leaky gut: mechanisms, measurement and clinical implications in humans. *Gut*.

[104] Suez J., Zmora N., Segal E., Elinav E. (2019). The pros, cons, and many unknowns of probiotics. *Nature Medicine*.

[105] Rao S. S. C., Rehman A., Yu S., Andino N. M (2018). Brain fogginess, gas and bloating: a link between SIBO, probiotics and metabolic acidosis. *Clinical and Translational Gastroenterology*.

[106] Żółkiewicz J., Marzec A., Ruszczyński M., Feleszko W (2020). Postbiotics-A step beyond pre- and probiotics. *Nutrients*.

[107] De Marco S., Sichetti M., Muradyan D. (2018). Probiotic cell-free supernatants exhibited anti-inflammatory and antioxidant activity on human gut epithelial cells and macrophages stimulated with LPS. *Evidence-Based Complementary and Alternative Medicine*.

[108] Chang P. V., Hao L., Offermanns S., Medzhitov R. (2014). The microbial metabolite butyrate regulates intestinal macrophage function via histone deacetylase inhibition. *Proceedings of the National Academy of Sciences*.

[109] Dimidi E., Christodoulides S., Scott S. M., Whelan K. (2017). Mechanisms of action of probiotics and the gastrointestinal microbiota on gut motility and constipation. *Advances in Nutrition: An International Review Journal*.

[110] Collins S. (1996). The immunomodulation of enteric neuromuscular function: implications for motility and inflammatory disorders. *Gastroenterology*.

[111] Cirillo C., Capasso R. (2015). Constipation and botanical medicines: an overview. *Phytotherapy Research*.

[112] Wang X., Yin J. (2015). Complementary and alternative therapies for chronic constipation. *Evidence-Based Complementary and Alternative Medicine*.

[113] Capasso F., Gaginella T. S., Grandolini G., Izzo A. (2003). Phytotherapy: a quick reference to herbal medicine. *Parasitology Research*.

[114] Bari M. S., Khandokar L., Haque E. (2021). Ethnomedicinal uses, phytochemistry, and biological activities of plants of the genus gynura. *Journal of Ethnopharmacology*.

[115] Cai Q. H., Ma R., Hu S. Y. (2018). A randomized controlled trial of Chinese patent medicine Xiao’er Biantong granules in the treatment of functional constipation in children. *Evidence-Based Complementary and Alternative Medicine*.

[116] Huang R. Y., Chen Y., Lu Y. F. (2016). Clinical observation on huangxin runchang pian in treating 45 cases of mild or moderate slow transit constipation. *Journal of Traditional Chinese Medicine*.

[117] Gong H., Qin F., He H. (2018). Herbal formula modified Buzhong-Yiqi-Tang for functional constipation in adults: a meta-analysis of randomized controlled trials. *Evidence-Based Complementary and Alternative Medicine*.

[118] Xu J., Lian F., Zhao L. (2015). Structural modulation of gut microbiota during alleviation of type 2 diabetes with a Chinese herbal formula. *The ISME Journal*.

[119] Tong X., Xu J., Lian F. (2018). Structural alteration of gut microbiota during the amelioration of human type 2 diabetes with hyperlipidemia by metformin and a traditional Chinese herbal formula: a multicenter, randomized, open label clinical trial. *mBio*.

[120] Xu J., Chen H.-B., Li S.-L. (2017). Understanding the molecular mechanisms of the interplay between herbal medicines and gut microbiota. *Medicinal Research Reviews*.

[121] Long C. X., He L., Guo Y. F. (2017). Effects of dendrobium candidum polysaccharide on immunity,intestinal microbiota and enzyme activity in mice with spleen deficiency constipation. *Natural Product Research and Development*.

[122] Liu D., Lin L., Lin Y. (2019). Zengye decoction induces alterations to metabolically active gut microbiota in aged constipated rats. *Biomedicine & Pharmacotherapy*.

[123] Yan S., Yang H. J., Yue Y. Z. (2017). Clinical observation and effect of tongbian decoction on laxative constipation and colon flora in elderly patients. *Chinese Journal of Experimental Traditional Medical Formulae*.

[124] Yan S., Yue Y. Z., Wang X. P. (2017). Aqueous extracts of herba cistanche promoted intestinal motility in loperamide-Induced constipation rats by ameliorating the interstitial cells of Cajal. *Evidence-Based Complementary and Alternative Medicine*.

[125] Wang W.-L., Xu S.-Y., Ren Z.-G., Tao L., Jiang J.-W., Zheng S.-S. (2015). Application of metagenomics in the human gut microbiome. *World Journal of Gastroenterology*.

[126] Klupczyńska A., Dereziński P., Kokot Z. (2015). Metabolomics in medical sciences - trends, challenges and perspectives. *Acta Poloniae Pharmaceutica-Drug Research*.

[127] Bashiardes S., Zilberman-Schapira G., Elinav E. (2016). Use of metatranscriptomics in microbiome research. *Bioinformatics and Biology Insights*.

[128] Verberkmoes N. C., Russell A. L., Shah M. (2009). Shotgun metaproteomics of the human distal gut microbiota. *The ISME Journal*.

[129] Chen F., Xue J., Zhou L., Wu S., Chen Z. (2011). Identification of serum biomarkers of hepatocarcinoma through liquid chromatography/mass spectrometry-based metabonomic method. *Analytical and Bioanalytical Chemistry*.

[130] Franzosa E. A., Sirota-Madi A., Avila-Pacheco J. (2019). Gut microbiome structure and metabolic activity in inflammatory bowel disease. *Nature Microbiology*.

[131] Sayin S. I., Wahlström A., Felin J. (2013). Gut microbiota regulates bile acid metabolism by reducing the levels of tauro-beta-muricholic acid, a naturally occurring FXR antagonist. *Cell Metabolism*.

